# Investigation of crack recognition and spatio-temporal evolution pattern in coal samples damage

**DOI:** 10.1038/s41598-023-45276-z

**Published:** 2023-10-20

**Authors:** Zeng Chen, Ping Wang, Feng Shi

**Affiliations:** 1 BGRIMM Technology Group, Building 23, Zone 18 of ABP, No. 188, South 4th Ring Road West, Beijing, 100160 People’s Republic of China; 2 China-South Africa Joint Research Center for Development and Utilization on Mineral Resources, Beijing, 102628 People’s Republic of China

**Keywords:** Engineering, Geophysics

## Abstract

Understanding the evolution mechanism of cracks helps to evaluate the behavior and performance of rock masses and provides a theoretical basis for the mechanism of crack propagation and instability. For this purpose, a rock mechanics testing system and an acoustic emission monitoring system were used to conduct acoustic emission positioning experiments on coal samples under uniaxial compression. According to clustering theory, the distribution pattern of microcracks and the dynamic evolution process of multiple cracks were studied. Subsequently, the reasons for the change in the spatio-temporal entropy (*H*) and fractal dimension (*D*) of a single crack were revealed. The research results show that microcracks present a statistical equilibrium distribution, the Gaussian distribution model is applicable to cluster crack distribution patterns, and a machine learning method can effectively identify cracks. The fractal dimension reflects the spatial characteristics of three-dimensional elliptical cracks, and low-dimensional cluster cracks are more likely to develop into macroscopic cracks. The change of *H* is related to the formation process of cracks, and an abnormal *H* (sudden increase and sudden decrease) could provide precursor information for the instability of coal samples. This research provides a new method to study crack distributions and formations and shows the competitiveness of the method in evaluating the damage state of coal.

## Introduction

In quasi-brittle materials such as rocks, concrete, and ceramics, the fracture strength distribution is usually determined by the size and spatial distribution of microcracks. The initiation, propagation, and coalescence of cracks are the main causes of rock failure and instability. Most rock failure processes are multi-scale evolution processes^1^ presented as the accumulation of multiple micro-damages, followed by growth of clusters and macro-scale catastrophe^2^.

Various monitoring methods have been used to study the damage process of rock masses under loading, including the digital image correlation technique^3–5^, computed tomography method^6–8^, infrared thermal imaging^9–11^, and ultrasonic technology^12,13^. Crack detection by two-dimensional fracture image recognition^14,15^, plane cluster analysis^16^, and static CT scan methods lacks crack spatio-temporal details. Promising results have been achieved in studies on cracks such as Brazilian splitting and pre-cracking^5,17–20^, however, the formation mechanism and evolution mode of multiple cracks are still unclear.

Rocks inevitably contain pores, joints, or microcracks. The appearance of spatio-temporal clustering can be considered an indication of the initiation of catastrophic fracture^21^. Clustering of seismic activity observed in Southern California has been proven to be able to identify foreshocks, mainshocks, and aftershocks^22,23^. Therefore, it is of great significance to study the distribution characteristics and formation mechanism of cluster cracks. The formation of planar clusters is mostly associated with failure at pre-existing weakened zones or the formation of new cracks in highly stressed zones due to stress redistribution^24^. Rock failure is determined by the weakest link. When the main crack begins to expand, the structure fails^25^. Crack information can be used to predict the strength of brittle materials. The investigation results show that the largest crack-cluster distribution under peak load follows a lognormal distribution^21^. In particular, if the defect cluster size distribution is described by the power law or exponent, the fracture strength follows Weibull distribution or Gumbel distribution^26,27^.

As a measure of system chaos, entropy is widely used in rock mechanics, seismology, nonlinear dynamics, and other fields. Research on dissipative structure theory shows that when an open system is far from equilibrium, the entropy can increase or decrease according to different conditions^28^. A precursor alarm is detected when an increase in the regional entropy of seismic information is followed by saturation or perturbation after the saturation^29^. There is obvious correlation between seismic entropy and tectonic state^30^, and this entropy method can be used to analyze seismic probability distributions^31^. Previous results have shown that entropy can serve as an applicable index for rock damage assessment^32,33^.

Acoustic emission (AE), a nondestructive monitoring method, can monitor the growth and activity of internal defects in samples, thus, it is widely used in rock mass damage analyses^34–38^. AE localization has clustering characteristics and a fractal structure in space or time^39^. From a hydraulic fracturing experiment, the spatial distribution of the AE events was clustered in the shape of an ellipsoid^40^.

In practical engineering scenarios, rock mass failure often involves the presence of multiple cracks. Interactions among these cracks can either facilitate or inhibit crack propagation. Under complex crack conditions, the distribution patterns of individual cracks have not been extensively studied, leading to insufficient criteria for assessing rock mass failure dominated by cracks. This study, through a statistical analysis of the distribution range of microcracks, introduces a method that employs a Gaussian mixture distribution model for crack identification. Furthermore, it investigates the variation patterns of entropy and fractal dimension of crack clusters during the coal sample failure process. The research findings offer a novel approach for discriminating coal sample damage.

## Experiment procedure

**Figure 1:**
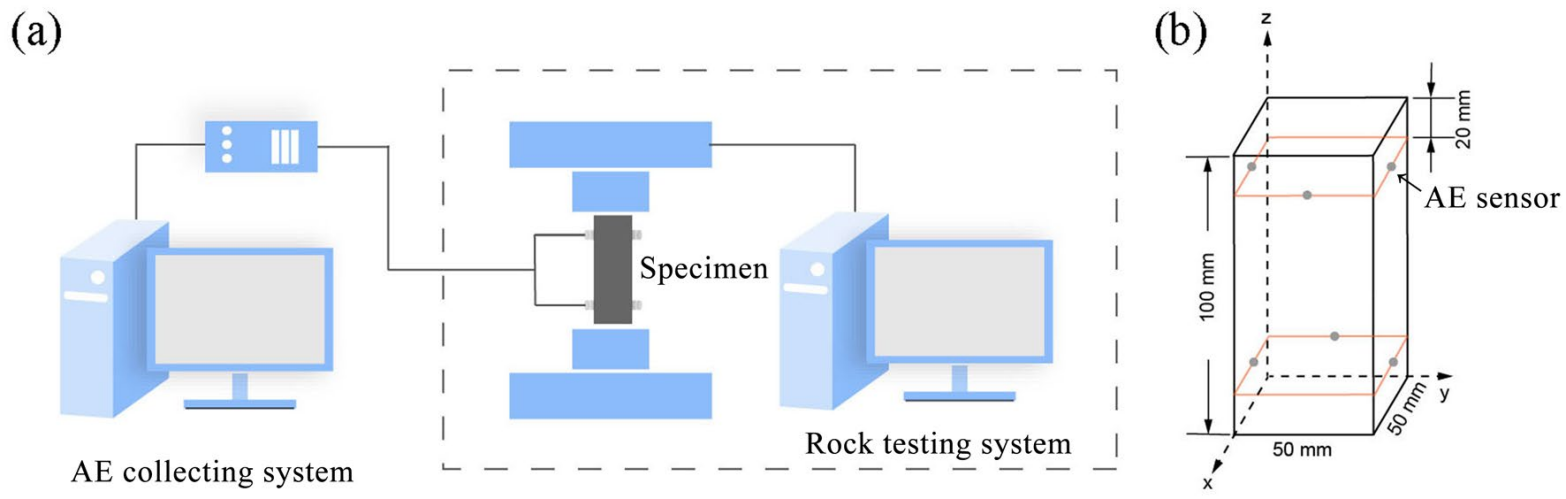
Experimental systems and AE sensor layout. (**a**) Experimental system. (**b**) Spatial arrangement of AE sensors.

In this study, the uniaxial compression test was carried out using the WDW-100E multi-function testing machine at the Beijing Computing Center, China. The PCI-II AE monitoring system of American Physical Acoustics Corporation (Fig. 1a) was used to obtain the AE signals related to the internal cracking events of coal samples. Coal sample drill is taken from the working face of the underground mine. In order to ensure the accuracy of the experiment, all samples were taken from the same coal mass, with axial deviation below 0.25° and flatness below 0.02 mm. Samples were processed into cuboids with side length of 50 mm and height of 100 mm.

Six Nano30 sensors with frequency sensitivities in the range 125–750 kHz and common amplifications (1220A-AST) of 40 dB were used in the experiment. The acquisition threshold was set to 45 dB, and the sampling rate was 1 MHz. AE sensor were glued to the surface of the specimen and uses petroleum jelly coupling contact surface. The sensor layout and placement are shown in Fig. 1b. The lead-off test was conducted before the experiment to ensure that all channels can receive signals stably. The sample was destroyed by loading at a speed of 0.2 mm/min by controlling the displacement. Table 1 shows the mechanical parameters of coal samples.

**Table 1:** Mechanical parameters of the coal specimens.

No	P-wave velocity (km/s)			Density (g/cm^3^)	Quality (g)	Poisson’s ratio	Uniaxial compressive strength (MPa)
	x	y	z				
F1	2.45	3.21	2.68	1.77	251.13	0.31	9.56
F2	1.86	1.91	1.85	1.51	252.32	0.30	6.25
F3	1.79	1.89	1.88	1.65	249.50	0.34	6.12
F4	1.97	1.73	1.86	1.47	245.66	0.33	7.64
F5	1.44	1.75	1.89	1.45	246.48	0.31	9.28

Pressure experiments were conducted on the samples using a rock testing system, while an AE collecting system collected AE signals in real-time during the experiments. Prior to the experiments, the physical and mechanical properties of each sample, especially the velocity information, were tested. This provided necessary references for the initial parameter configuration of the AE system. To ensure continuous monitoring, the AE data collection system was synchronized with the rock testing system during startup.

## Theoretical basis

### Clustering theory

In the case of thousands of microcracks, the application of machine learning for crack identification shows advantages in speed and accuracy. Through machine learning, the cluster crack distribution can be identified. In this study, five typical clustering algorithms were selected to study the distribution of cracks considering the distance, similarity, and calculation model.

K-means is a distance-based clustering algorithm, that is, the closer the distance between objects is, the greater the similarity between them. The algorithm identifies the objects with a compact distance as independent clusters. The distinguishing factor between K-medoids and K-means algorithms is the method for determining the center point. K-medoids takes the point with the minimum distance between each class point and other points as the center, whereas K-means takes the mean of each class point as the center. Fuzzy C-means (FCM) clustering is a partition-based clustering algorithm that maximizes the similarity between objects that are divided into the same cluster and minimizes the similarity between different clusters.

Hierarchical (HAC) clustering^41^ creates a hierarchical nested clustering tree by calculating the similarity between data points of different categories. Hierarchical clustering methods include single-linkage, complete-linkage, and average-linkage. The single-link algorithm used in this study is based on the minimum distance.

The gaussian mixed model (GMM) is a weighted combination of multiple Gaussian distribution models and uses the expectation–maximization (EM) algorithm for iteration to fit any type of distribution. A comparison between the GMM and K-means algorithm showed that K-means had a poor clustering effect for data with small samples and non-circular shapes, and it had poor clustering effect for unbalanced sample categories.

### Information entropy

Entropy was originally presented as a thermodynamics concept. It is a physical quantity that indicates the disorder and chaos degree of a molecular system. If the entropy is large, the chaos degree of the system is large, whereas if the entropy is small, the chaos degree of the system is small. Assuming a distribution over the occurrences of possible outcomes, ${\lambda}= \left( {\lambda_{1} ,\lambda_{2} , \ldots ,\lambda_{n} } \right)$, the following two conditions are met:

1 $$0 \le \lambda_{i} \le 1\;\left( {i = 1,2, \cdots ,n} \right)$$

2 $$\mathop \sum \limits_{i = 1}^{n} \lambda_{i} = 1$$

3 $$H\left( X \right) = H\left( {\lambda_{1} ,\lambda_{2} , \ldots ,\lambda_{n} } \right) = - k\mathop \sum \limits_{i = 1}^{n} \lambda_{i} \log \lambda_{i} \;\left( {k \ge 0} \right)$$

In the sample system, the variation of the spatio-temporal entropy (*H*) reflects the degree of uniformity of the microcrack distribution. Through a fixed time period and sliding with a certain space step, the microseismic activity entropy can be scanned in space. The calculation process of micro-crack spatio-temporal entropy (*H*) is as follows:

1. The cells on the sample space are divided and the unit time length is determined.
2. The proportion of the number of AE events in each cell per unit time is calculated.
3. The crack spatio-temporal entropy of the loading process is calculated based on the information theory.

## Results and analysis

### Evaluation of clustering result

Cluster analyses were conducted on samples under different cluster numbers and the results were evaluated. The contour coefficient was measured quantitatively by comparing the similarities between all data sets and was used to evaluate the clustering recognition results. The silhouette coefficient (SC) represents the average value of the silhouette coefficients of all samples and is within the range [− 1, 1]. The higher the matching between a sample and its cluster, the larger the SC value, indicating a better the clustering result. Due to the significant differences in the monitoring process data of sample F3 compared to the others, this aspect is not discussed in this paper. Letters (a)–(d) in the figures represent specimens F1, F2, F4, and F5, respectively.

**Figure 2:**
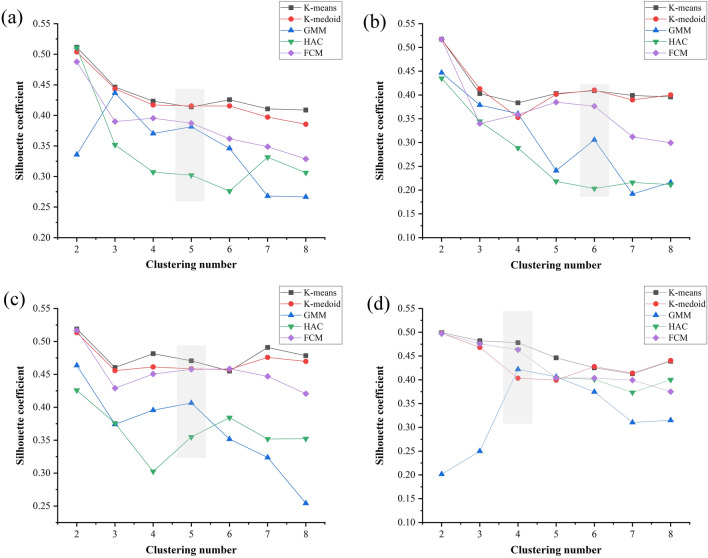
Silhouette coefficients for clustering methods under different number of clusters. (**a**)–(**d**) represents F1, F2, F4, F5 samples.

The analysis results show that the similarity between each data sample slightly decreases with an increase in the number of samples, as shown in Fig. 2. The effect of multiple clustering shows a decreasing trend with an increasing number of clusters. K-medoids, K-means, and FCM exhibited high stability in their recognition effect and always maintained high silhouette coefficients. HAC uses the minimum distance to determine the cluster, which shows limitations in this application scenario. In particular, GMM is sensitive to the setting of the number of clusters. For different numbers of samples, GMM considers the weight of each class in the calculation process and outputs the classification results in the form of a probability distribution. GMM are usually based on random initialization parameters, which means that different runs can lead to different results. If the number of clusters selected does not match the actual structure of the data, overfitting or underfitting may result. When the data itself presents a complex distribution in some regions, more Gaussian distributions are needed to fit the data, so the number of clusters needs to increase. The variation of the SC also reflects the heterogeneity among clusters (cracks). In general, the optimal clustering effect does not represent the final crack position. Therefore, it is necessary to set the optimal number of clusters. The principle is to distinguish as many clusters as possible so that the sample retains more characteristic information. When selecting the optimal number of clusters, in cases where the SC doesn’t vary significantly, it is often advisable to opt for a larger number of clusters. This allows for a more detailed representation of complex crack distribution patterns. By comparing the recognition effects of all the methods, the optimal cluster number of the corresponding sample at the shadow mark in Fig. 2 was obtained.

### Crack distribution model

**Figure 3:**
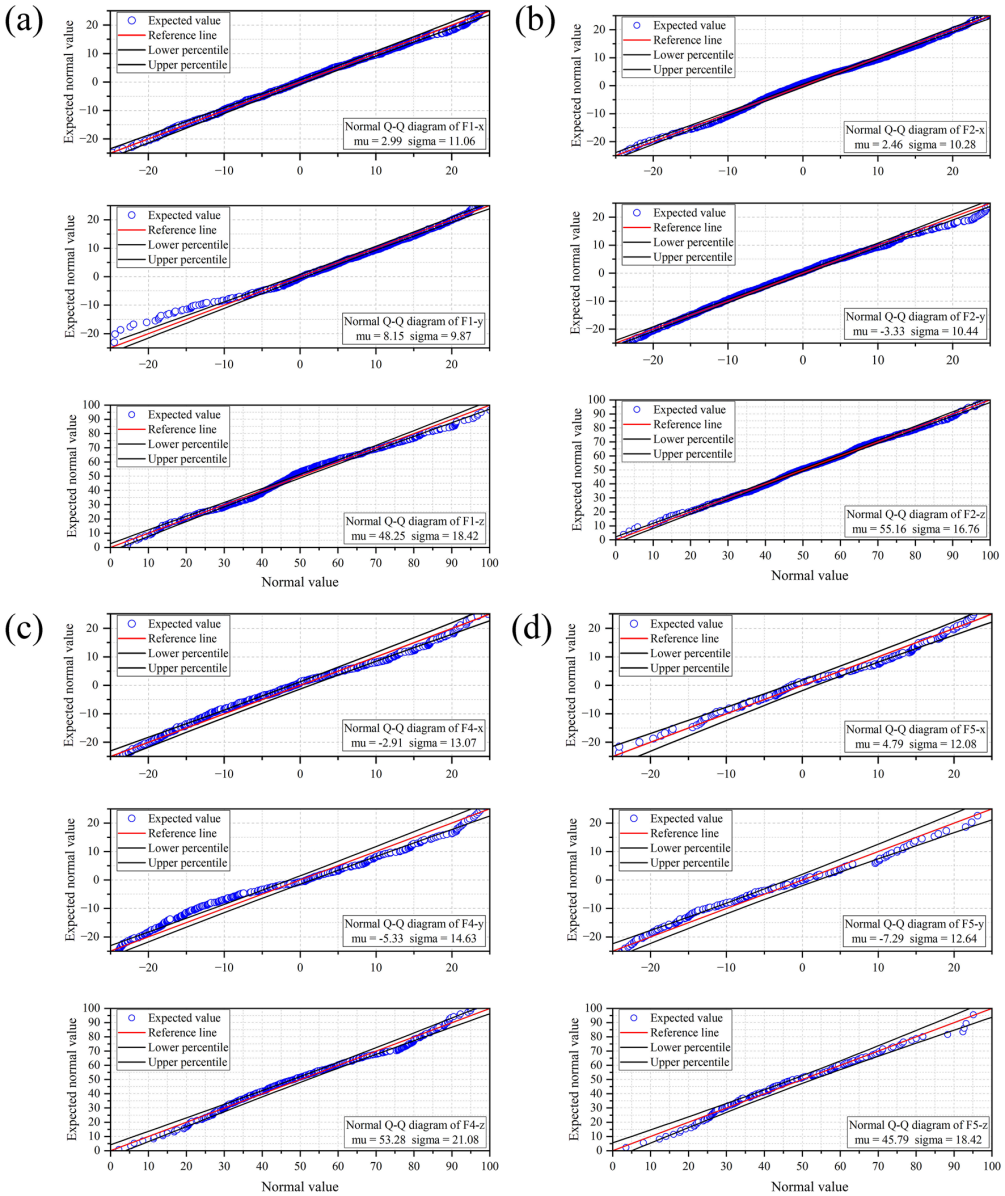
Q–Q plots of Gaussian distribution. (**a**)–(**d**) represents F1, F2, F4, F5 samples.

Compared with other mathematical models, we found that the Gaussian distribution could better characterize the spatial distribution of microcracks. As shown in Fig. 3, Q–Q plots compare a quantile of test sample data with a known distribution to a test data distribution. The spatial location distribution of microcracks in three axial directions of all samples conforms to the Gaussian distribution, which indicates that these events are independent and random. The density function of the microcrack probability distribution can be expressed as:

4 $$f\left( x \right) = \frac{1}{{\sqrt {2\pi } \sigma }}\exp \left( { - \frac{{(x - \mu )^{2} }}{{2\sigma^{2} }}} \right)$$

The microcracks are symmetrically distributed, with high density in the middle and gradually decreasing density towards the peripheral. Expected (center) values of the F1, F2, F4, and F5 microcracks in the x-axis (μ) are 2.99, 2.46, − 2.91, and 4.79; the expected (center) values in the y-axis are 8.15, − 3.33, − 5.33, and − 7.29; and the expected (center) values in the z-axis are 48.25, 55.16, 53.28, and 45.79, respectively. The analysis results show that the microcracks are mainly concentrated in the middle area of the sample in the x- and z-axis directions but deviate from the center of the y-axis. The magnitude of sigma reflects the degree of concentration of the microcracks with respect to the center. Sixty eight percent of the microcracks were concentrated in approximately seventeen percent of the space. The distribution characteristics of microcracks reflect the distribution patterns of strength throughout the rock, which are related to the inherent fractures and stress distribution within the rock mass.

### Crack recognition results

In various stages of the strength tests, a series of AE events related to space and time are accumulated into clusters. Clustering may indicate the formation of macroscopic crack planes, which indicates that the critical state is nearly reached. When the time interval of events is short, the distribution converges toward a Gaussian distribution. The EM algorithm is used to calculate the spatial distribution of a single crack. The estimated parameter value was used to calculate the log-likelihood expectation, and the parameters were updated by maximizing the log-likelihood expectation to obtain each distribution model.

Assuming that $\overline{X}$ and $S$ are the sample mean vector and covariance matrix for $n$ samples with 3 variables, a 100(1−α)% confidence ellipsoid can be calculated using *Hotelling’s T-squared* (T^2^) distribution:

5 $$(X - \overline{X} )^{'} S^{{ - 1}} \left( {X - \overline{X} } \right) = \frac{{3\left( {n - 1} \right)}}{{\left( {n - 3} \right)}}F^{\text{'}}inv\left( {1 - \alpha ,3,n - 3} \right)$$

where $F^{\text{'}}inv$ is the inverse F cumulative distribution function.

The 100(1−α)% prediction ellipsoid can be expressed as:

6 $$(X - \overline{X} )^{'} S^{{ - 1}} \left( {X - \overline{X} } \right) = \frac{{3\left( {n + 1} \right)\left( {n - 1} \right)}}{{\left( {n - 3} \right)n}}F^{\text{'}}inv\left( {1 - \alpha ,3,n - 3} \right)$$

**Figure 4:**
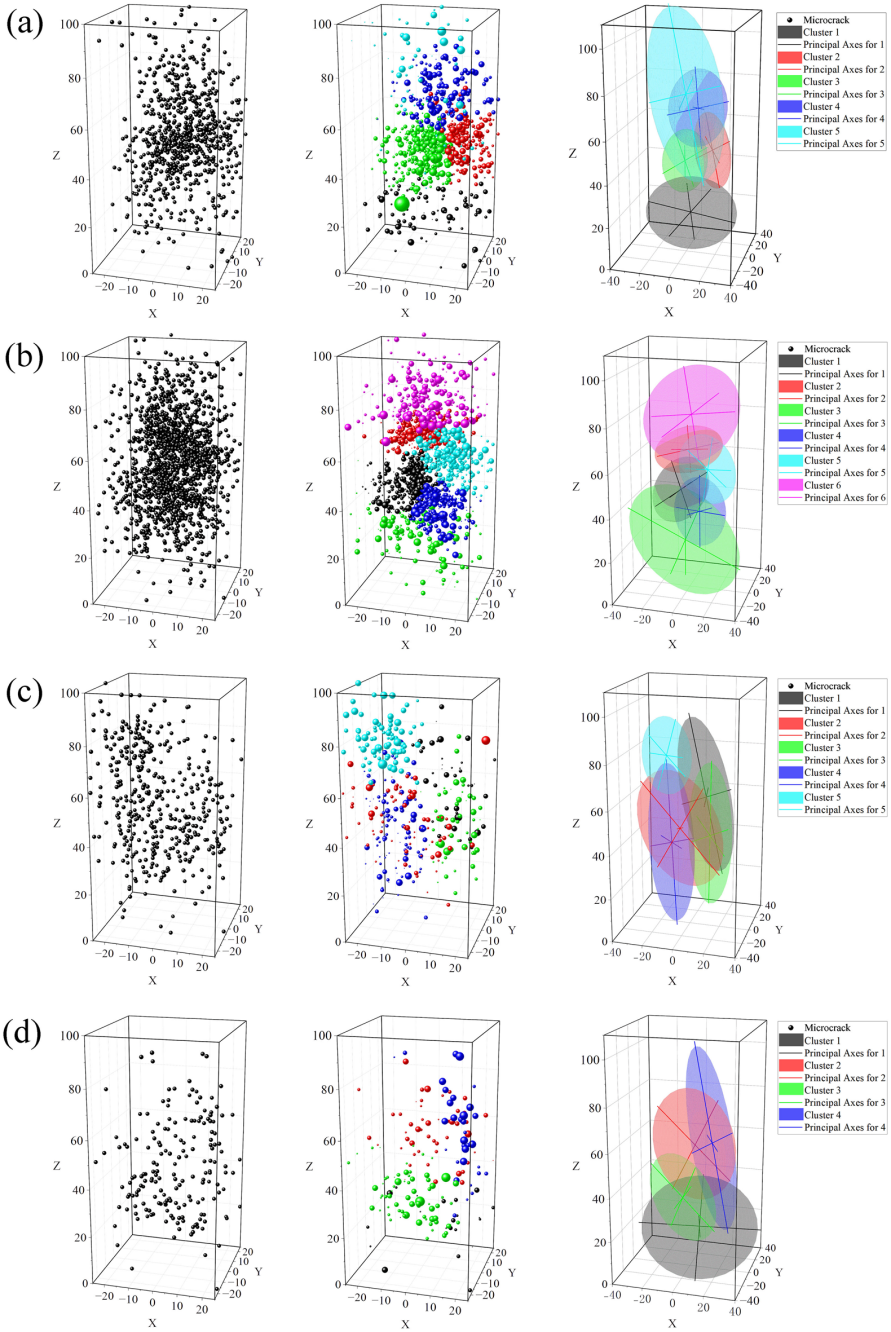
Cluster cracks and distribution of confidence intervals. (**a**)–(**d**) represents F1, F2, F4, F5 samples. The original microcrack distribution, microcrack classification and macroscopic single crack are represented from left to right.

By calculating the confidence interval of different cracks, we characterize an ellipsoid for which the seismicity is clustered through the principal axes (a, b, c). The length of the main axis and the direction of the first main axis (a) of the ellipsoid are listed in Table 2. Figure 4 shows the crack identification results, namely, the original microcrack distribution, microcrack classification, and macroscopic single crack characterization, where the cracks are distinguished by different colors. To reduce the impact of energy, the logarithm of energy was used to map the size of microcracks.

**Table 2:** Parameters of single crack in ellipsoid.

No	Clusters	Principal semi-axial (mm)			Slope of the tangent line (First principal axis)		
		a	b	c	x	y	z
F1	1	33.91	23.74	14.74	-0.94	0.35	-0.02
	2	19.59	14.77	11.53	0.38	-0.58	-0.72
	3	19.78	15.41	19.98	-0.89	-0.21	-0.40
	4	20.68	17.97	8.67	0.13	-0.05	-0.99
	5	41.95	23.13	18.44	-0.81	-0.57	-0.09
F2	1	27.61	18.19	13.74	-0.05	0.99	-0.10
	2	23.94	18.15	10.39	-0.59	-0.81	-0.01
	3	38.03	25.23	21.24	-0.81	-0.39	0.42
	4	18.67	15.47	11.86	-0.91	0.40	-0.07
	5	23.46	16.01	14.28	-0.29	-0.81	0.49
	6	31.36	29.14	20.51	-0.92	0.37	-0.13
F4	1	39.23	15.89	10.59	-0.22	-0.22	0.95
	2	30.63	21.54	9.36	-0.79	0.05	0.60
	3	35.59	18.72	12.44	-0.88	0.27	-0.96
	4	37.17	23.36	13.96	-0.19	0.35	0.91
	5	19.45	15.93	12.34	-0.75	0.59	0.31
F5	1	38.38	29.72	8.54	-0.99	-0.17	0.01
	2	30.39	23.61	15.83	-0.62	-0.49	0.61
	3	23.99	18.93	10.48	-0.883	0.06	0.55
	4	45.17	12.89	9.99	-0.16	-0.27	0.95

Generally, the number of optimal clusters is proportional to the number of events. The Gaussian model is not applicable when the number of microcracks is small, which is caused by the fact that the sample data does not match the number of calculation models. A number of cluster cracks are concentrated in the middle of the sample, and their distribution ranges overlap. From the analysis results, it can be assumed that the ellipsoidal-shaped cluster of hypocenters can be used to characterize the spatial extension of the main fracture assuming the area.

The length of the first principal semi-axial (*a*) of the middle crack in F1 (794 microcracks) and F2 (1491 microcracks) is approximately 20 mm. The microcracks in F4 (410 microcracks) and F5 (181 microcracks) are fewer than those in F1 and F2, the distribution is scattered, and the length of the first principal semi-axial (*a*) generally exceeds 30 mm. When the number of microcracks varies greatly, the crack angle and its distribution range remain consistent. The principal axis of the confidence interval can indicate the crack development direction. The high-energy AE events are mostly concentrated around the cluster cracks, that is, the energy value at the crack intersection is large.

### Crack initiation stress and propagation direction

Griffith’s criterion proposes that the increase in surface energy is balanced by the decrease in the elastic energy stored in the specimen as the crack grows. For a crack that is oblique to the principal stress, the crack orientation is derived to obtain the extreme value, and the local maximum tensile stress can be expressed as follows:

7 $$A{\sigma }_{\theta }=-\frac{({\sigma }_{1}-{\sigma }_{3}{)}^{2}}{4({\sigma }_{1}+{\sigma }_{3})}$$

where ${\sigma }_{\theta }$ is the maximum tangential tensile stress of the defect side wall and $A$ is the ratio of the short axis to the long axis of the elliptical crack. When the crack is perpendicular to the direction of principal stress, $A{\sigma }_{\theta }=2{\sigma }_{3}$.

The failure condition of a rock under uniaxial tension satisfies the condition $A{\sigma }_{\theta }-2{\sigma }_{t}=0$, and substituting this condition in Eq. (7) yields the following result.

8 $$(\sigma_{1} - \sigma_{3} )^{2} = 8\sigma_{t} \left( {\sigma_{1} + \sigma_{3} } \right)$$

Cracks are closed under a confining pressure. Thus, considering the friction between crack walls after crack closure, the maximum local tensile stress takes the following form:

9 $$A{\sigma }_{\theta }=-\frac{\sqrt{1+{\nu }^{2}}-\nu }{2}({\sigma }_{1}-{\sigma }_{3})+\nu {\sigma }_{3}$$

where $\nu$ is the friction factor between cracks.

In practice, the cracks inside a rock cannot be completely merged, and the actual local tensile stress should be in the range between Eqs. (7) and (9).

**Figure 5:**
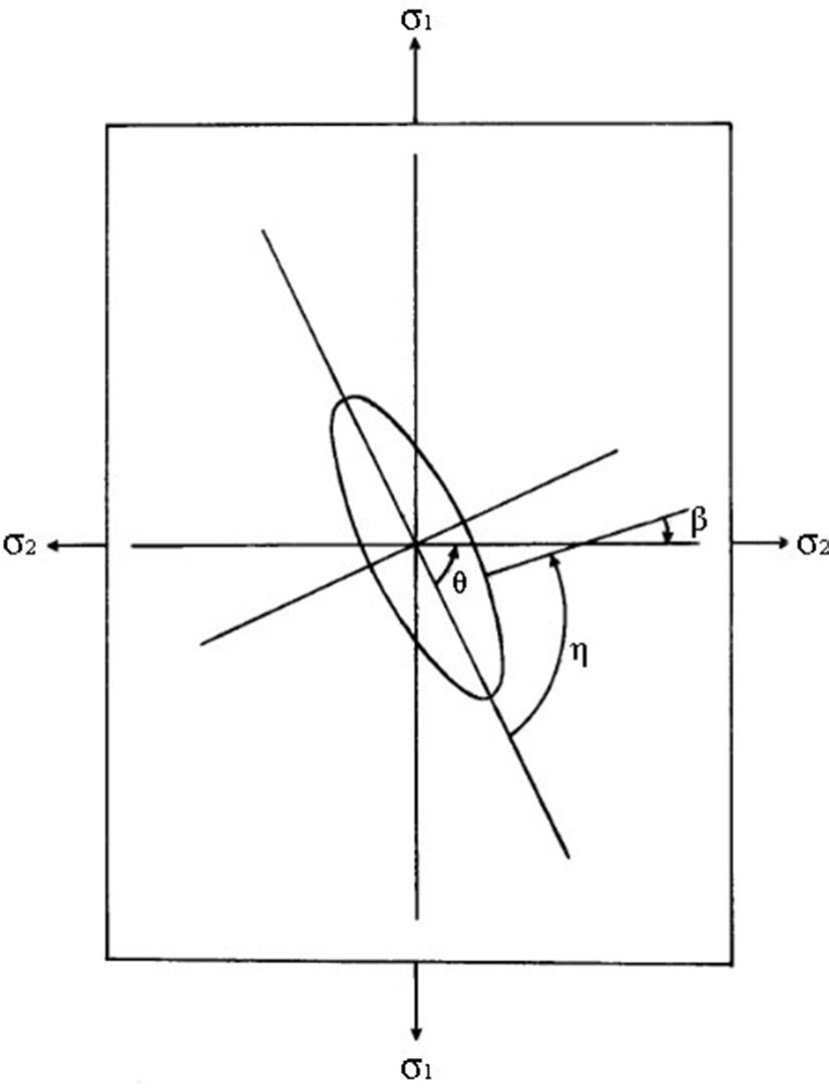
Elliptical crack and angles involved.

In general, Griffith’s theory does not provide information on the rate or direction of fracture propagation. Figure 5 shows the angles involved in the macro fracture standard definition. The crack angle satisfies the following formula:

10 $$\beta =\eta -\theta$$

where $\beta$ is the direction of the normal of the crack circumference relative to the secondary direction and $\theta$ is the angle between the major axis of the elliptic curve and the secondary stress ($\sigma_{2}$).

11 $$\left\{\begin{array}{c}\eta ={\mathit{tan}}^{-1}\left[\frac{{\sigma }_{1}{\mathit{sin}}^{2}\theta +{\sigma }_{2}{\mathit{cos}}^{2}\theta -({\sigma }_{1}^{2}{\mathit{sin}}^{2}\theta +{\sigma }_{2}^{2}{\mathit{cos}}^{2}\theta {)}^\frac{1}{2}}{({\sigma }_{1}-{\sigma }_{2})\mathit{sin}\theta \mathit{cos}\theta }\right]\\ \eta =0\\ \eta ={\mathit{tan}}^{-1}\left[-\frac{2{\sigma }_{c}+(4{\sigma }_{c}+{\sigma }^{*2}{)}^\frac{1}{2}}{{\sigma }^{*}}\right]\end{array}\right.$$

where $\sigma_{c}$ is the stress required to close the crack (in the direction perpendicular to that of the stress the crack without friction), and:

12 $${\sigma }^{*}=({\sigma }_{1}-{\sigma }_{2})\mathit{sin}2\theta +\mu ({\sigma }_{1}+{\sigma }_{2}-2{\sigma }_{c}-({\sigma }_{1}-{\sigma }_{2})\mathit{cos}2\theta )$$

There are a large number of micro-defects in rocks. Under stress concentration, these defects can easily initiate microcracks, which in turn expand and connect to form macro fracture. The crack initiation is mostly concentrated around the microstructure surface. Through a theoretical analysis of the crack propagation direction, the crack propagation directions under three different conditions can be obtained. At $\eta = 0$, the crack propagation is along the direction of the original crack. In other cases, the crack will develop in the form of branch cracks. Cracks are formed in sequence and tend to be propagate in the direction of the larger compressive stress.

Theoretical analysis can support the identification results. As shown in Fig. 4 and Table 2, most cracks formed up and down in the sample, and the angles of multiple crack clusters tilted in the same direction. In the sample, the development direction of crack clusters with close center distance is similar (e.g. cracks 4, 5 in F1; cracks 3, 4 or 1,2 in F2; cracks1, 3 or 2, 4 in F4; cracks 2, 4 in F5).

## Discussion

The crack identification method proposed in this paper, through the analysis of continuous monitoring data, can significantly enhance the accuracy and precision of identifying internal cracks in coal and rock formations. Due to the complexity of real-world engineering scenarios, the instability and failure of rock masses dominated by cracks are often influenced by the superposition of multiple cracks, leading to unclear instability criteria. Through the use of single-crack identification techniques, it becomes possible to effectively clarify the instability criteria.

### Crack Spatio-temporal evolution features

**Figure 6:**
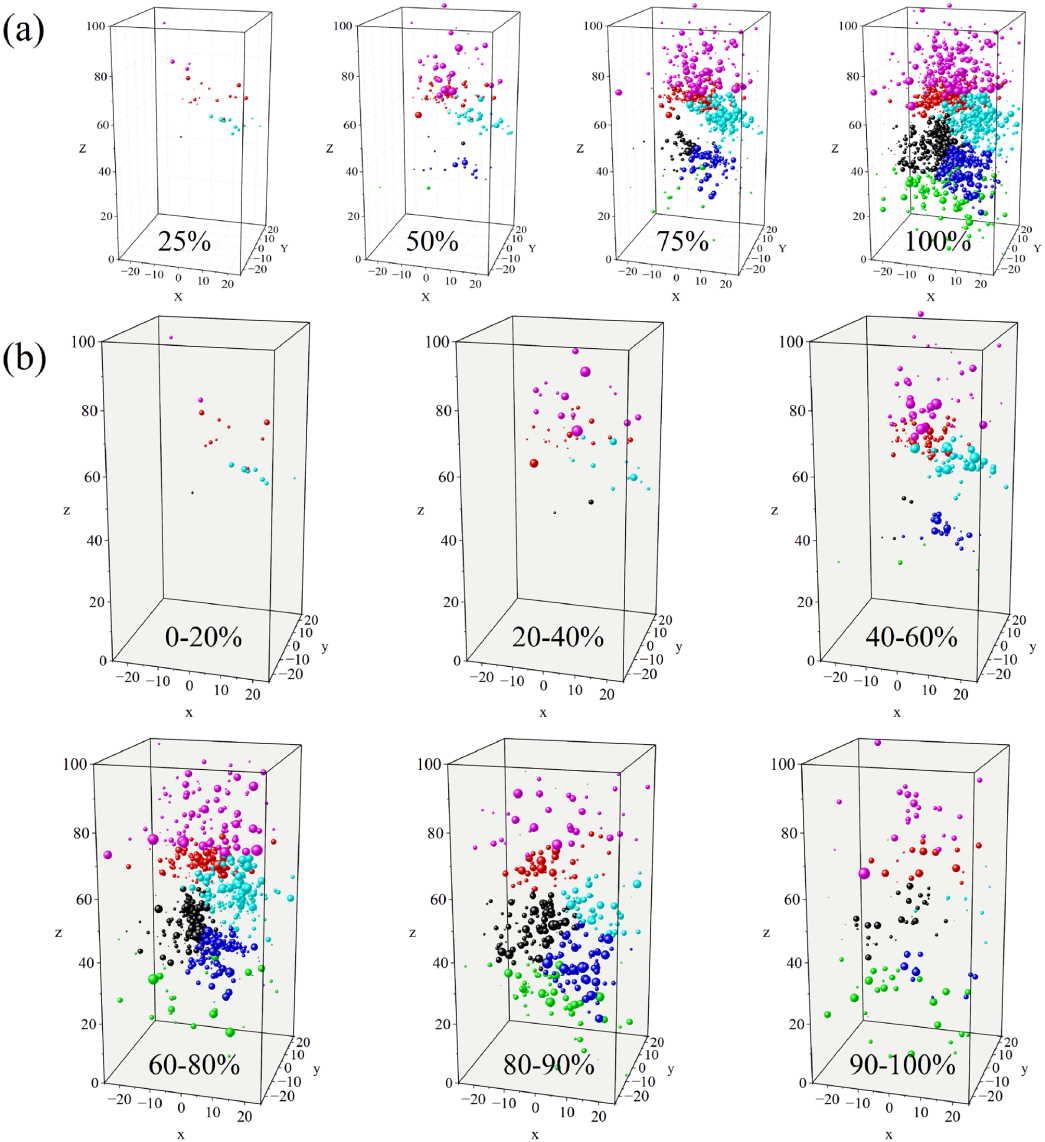
Temporal and spatial distribution of cracks in F2. (**a**) Distribution of microcracks at a certain stage. (**b**) Distribution of microcracks in different percentage stages.

Based on the percentage of test time, Fig. 6a shows the development of microcracks at each stage for the F2 sample. The microcracks are first generated from the upper part of the sample, and the cluster crack position is relatively independent. As the experiment progressed, a blank area was formed in the middle of the sample, which was most noticeable at the 75% stage. Notably, the crack did not continue to grow, but the density of microcracks in the center increased continuously and propagated towards the crack tip. The cluster cracks expanded and connected around the middle nucleation region, and the propagation mode was similar to an “onion petal”, with the same center but different deflection of the propagation direction.

Presently, there is no effective method to monitor all the cracks caused by the instability of the specimen. As shown in Fig. 6b, during the initial stage (0–20%), AE signals generated in the initial crack closure stage are mainly caused by the friction and closure of the initial crack. The extent of this nonlinear section depends on the crack density and geometric characteristics of the crack population. The microcracks are concentrated in the 60–90% stage, and the high energy events increase.

Microcracks clusters macroscopically determine the location of stable cracks. For areas with relatively low strength, the accumulation of energy leads to the growth and interaction of many microcracks. For areas with high strength, the accumulated energy is insufficient to cause many microcracks. Cracks in the process of rock instability consisted of three main processes: divergence in the linear deformation stage, convergence and concentration during the initiation of microcracks, and relative disorder after the formation of macrocracks.

### Theoretical basis of information entropy

**Figure 7:**
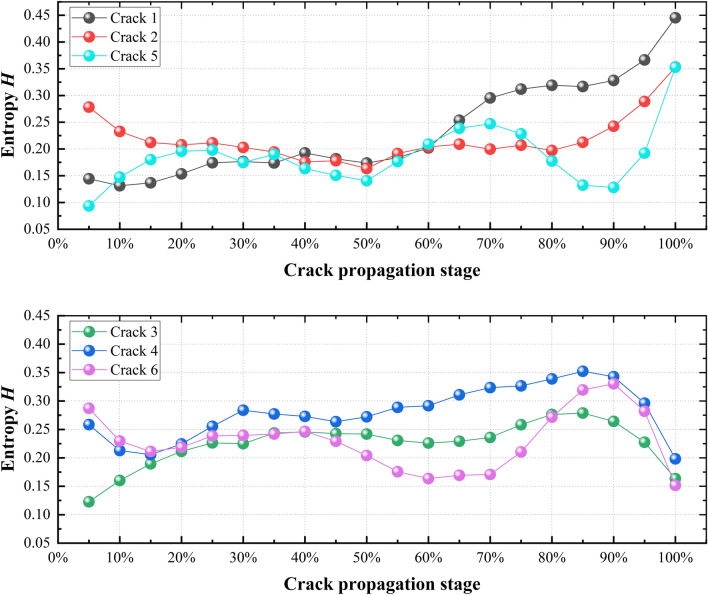
Spatio-temporal entropy (*H*) characteristic of single-crack in F2.

Using the spatio-temporal entropy calculation method mentioned in Sect. 3, we calculated the identified crack clusters, and the results are shown in Fig. 7. At the initial loading, the initial crack is closed and the entropy of the microcrack decreases (i.e., cracks 1 and 6). When the crack initiation stress is reached, micro-crack initiation occurs, but the number of micro-cracks is small and the entropy fluctuation is low. After the further development of microcracks, the entropy of cracks 1, 2, and 5 increases after 50%, and the entropy of cracks 3, 4, and 6 increases after 60%. In the late stage of sample failure (90–100% stage), there is a sudden increase or decrease in information entropy (*H*) appears a sudden increase or decrease.

The entropy fluctuation reflects the change of the propagation direction of the microcrack. The location uncertainty of microcrack initiation leads to a variety of combination modes, which are represented by a high information entropy and low aggregation degree in space. In contrast, the uncertainty of microcrack initiation is small, which is represented by a small information entropy and high aggregation degree in space. A sudden increase or decrease in entropy is considered to indicate the initiation of catastrophic fracture. The surge indicates that the location of the microcrack is changed from concentrated to pointed (peripheral), which may indicate that the cracks are intersecting. The sudden decrease in entropy indicates the microcrack in the middle, which implies that the microcracks are connected and the crack is formed. In the failure stage of the sample, the time of initiation of a single crack and the speed of development are not the same. By calculating the entropy change of a single crack, the cross influence can be reduced as much as possible and the crack evolution law can be revealed more accurately.

### Dimension of crack plane

The dimension of the crack plane can further characterize the complexity of a crack and provide the basis for evaluating the stable state of the crack. The larger the dimension, the higher complexity, and the more information it contains. A decrease in the fractal dimension indicates a decrease in the complexity of the microcrack distribution and the imminence of danger. The dimensionality of macroscopic cracks decreased, which implies that the microcracks were concentrated in one surface or in a small range. There is no simple linear relationship between the size of the fractal dimension and the number of microcracks, but the fractural dimension mainly depends on the spatial location distribution. In the same crack cluster, the fractal dimension of different planes will also appear a big difference. When each event is independent and random, dimension reduction is usually accompanied by the generation of a broken section.

**Figure 8:**
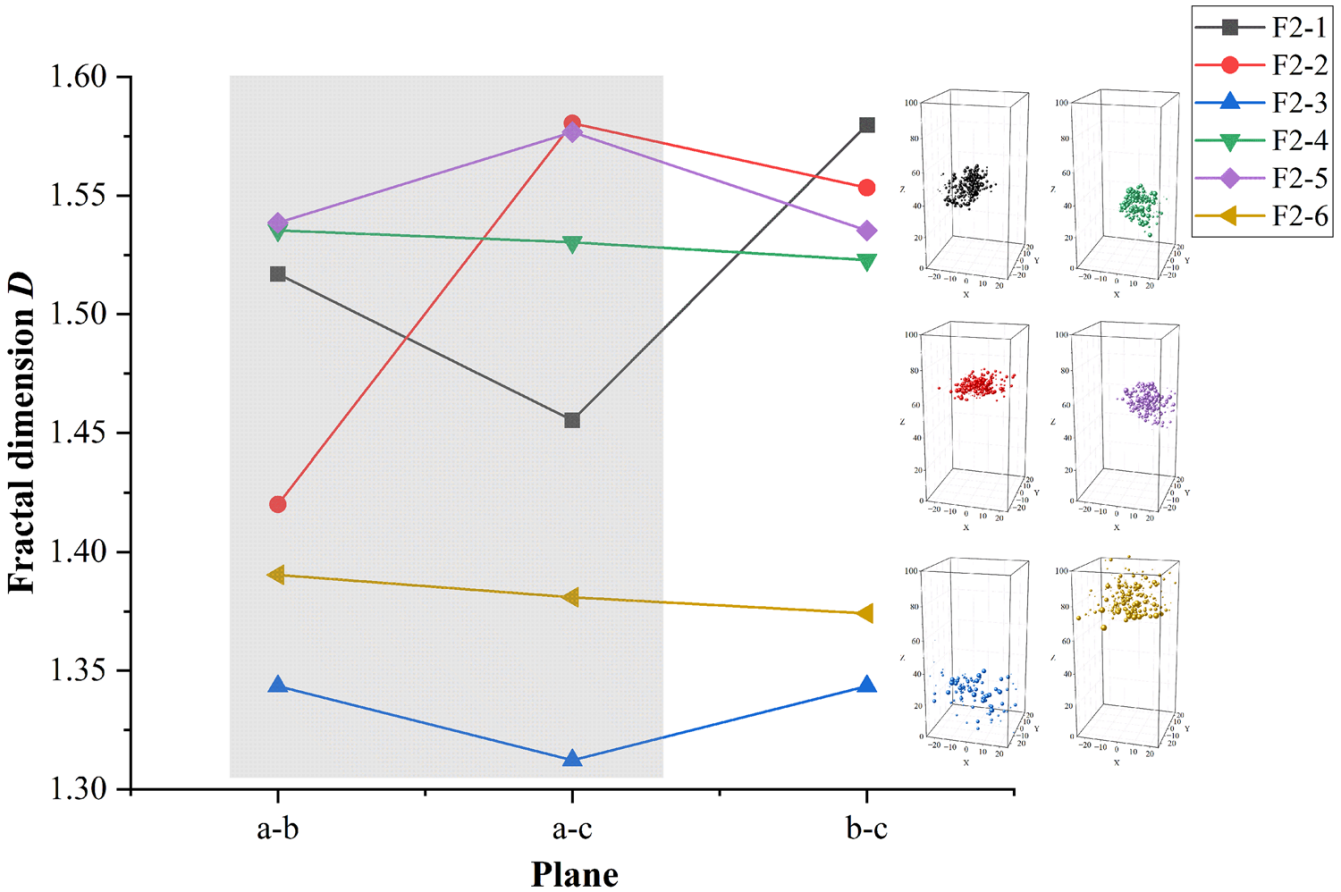
The fractal dimension of single-crack.

In Fig. 8a–c are the two main planes characterizing the ellipsoid shape. From the analysis results, the fractal dimension of one of the surfaces of cracks 1, 2, and 5 is small, while the fractal dimensions of cracks 3, 4, and 6 are almost the same. The former type of crack converges and tends to concentrate into a plane, while the latter type of distribution is more dispersed. The analysis results show that the fractal dimension and entropy change patterns are consistent, and cracks 1, 2, and 5 and cracks 3, 4, and 6 are divided into two types of cracks with different characteristics for the same change in entropy and fractal dimension. We believe that under the condition of a sudden increase in spatio-temporal entropy and large difference in the plane fractal dimension in the failure stage, the crack is expanded under the action of a peak load, and under the condition of a sudden decrease in the entropy and small difference in the plane fractal dimension in the failure stage, the crack is manifested as the combination of microcracks.

## Conclusion

In this study, the spatial distribution pattern of cracks is analyzed, and a crack identification and characterization method is proposed. The information entropy and fractal dimension are used to analyze the spatio-temporal evolution of cracks, providing a theoretical basis for the crack propagation and instability mechanism. Based on this study, the following conclusions can be drawn.

1. Under the condition of uniaxial compression, the unstable crack of the coal sample presents a statistical balanced distribution, which conforms to the Gaussian model, and the crack distribution is symmetrical, with a high density in the middle and gradual decrease in density toward the peripheral.
2. Instead of continuous crack propagation, the density of the micro crack in the center increased continuously and expanded towards the crack tip simultaneously. The propagation mode was similar to the “onion flap,” with the same center but different deflection of the propagation direction.
3. The entropy fluctuation reflects the propagation direction of the microcrack to a certain extent. The information entropy is high and the aggregation degree is low in space. In contrast, the information entropy is small and the aggregation degree is high in the space range. The sudden increase and decrease of entropy may be the precursor of crack instability.
4. The fractal dimension reflects the spatial evolution characteristics of three-dimensional elliptic cracks, and low dimensional cluster cracks are more likely to develop into macroscopic cracks. The failure of the sample is the result of the combined effect of crack propagation under the action of peak load and smaller cracks.

## Data Availability

The datasets generated during and/or analysed during the current study are available from the corresponding author on reasonable request.
